# ICIs Exceptional Long Response in TNBC: Addressing the Issue of Optimal ICIs Duration. Two Cases and Review of the Literature

**DOI:** 10.1002/cnr2.70397

**Published:** 2025-11-07

**Authors:** Simone Rota, Carolina Sciortino, Silvia Damian, Matteo Duca, Giorgia Villa, Matteo De Monte, Elisabella Ebrahem, Laura Cattaneo, Chiara Volpi, Alessandra Casale, Diletta Sorrentino, Sara Pessina, Antonia Martinetti, Filippo De Braud, Sara Cresta

**Affiliations:** ^1^ Medical Oncology Department Fondazione IRCCS Istituto Nazionale dei Tumori di Milano Milan Italy; ^2^ Department of Pathology and Laboratory Medicine, First Division of Pathology Fondazione IRCCS Istituto Nazionale dei Tumori Milan Italy; ^3^ Department of Interventional Radiology Fondazione Istituto di Ricovero e Cura a Carattere Scientifico (IRCCS) Istituto Nazionale Dei Tumori Milan Italy; ^4^ Department of Oncology and HematoOncology University of Milan Milan Italy

**Keywords:** breast cancer, immune checkpoint inhibitors, immunotherapy, systemic anticancer treatment, triple negative breast cancer

## Abstract

**Background:**

Breast cancer is the leading cause of cancer‐related mortality in women, with triple‐negative breast cancer (TNBC) being an aggressive subtype associated with poor prognosis and limited treatment options. TNBC is known for its immunogenic characteristics, including high genetic instability and elevated tumor‐infiltrating lymphocytes (TILs). Immune checkpoint inhibitors (ICIs) have shown efficacy in TNBC treatment, but the optimal treatment duration in case of prolonged response remains unclear.

**Case:**

This case series here reported presents two patients with metastatic TNBC who demonstrated an excellent response to ICI therapy. The first patient, a 60‐year‐old, was enrolled in a Phase I clinical trial and received a combination of anti‐PD‐1, anti‐LAG‐3, and anti‐CSF‐1 monoclonal antibodies. The second patient, a 45‐year‐old with BRCA1‐mutated TNBC, participated in a Phase II trial and received a combination of avelumab (anti‐PD‐L1) and talazoparib (PARP inhibitor). Both patients achieved a complete radiological response (CR), which has been maintained for 5 years. A literature review performed here identified seven additional long‐term ICI responders in metastatic TNBC. While ICIs showed significant efficacy in some patients, variability in PD‐L1 expression and TILs suggests that other factors may influence response. Two patients in previous studies discontinued ICIs after 2 years without progression, prompting questions about the optimal treatment duration.

**Conclusion:**

ICIs optimal treatment duration remains uncertain. Literature on metastatic melanoma suggests that discontinuing ICIs after a complete response rarely leads to recurrence. Prospective studies and emerging biomarkers, such as circulating tumor DNA, may help tailor treatment decisions.

**Abbreviations**
BRCA: breast cancer gene
CPS: combined positive score
CR: complete response
CSF1: colony‐stimulating factor 1
CT: computed tomography
CTLA‐4: cytotoxic T‐lymphocyte–associated protein 4
DFI: disease‐free interval
EC: epirubicin + cyclophosphamide
FEC: 5‐fluorouracil + epirubicin + cyclophosphamide
GE junction: gastroesophageal junction
HNSCC: head and neck squamous cell carcinoma
IC: immune score
ICI: immune checkpoint inhibitor
IHC: immunohistochemistry
LAG‐3: lymphocyte activation gene 3
MMR: mismatch repair
MSI: microsatellite instability
NA: not available
NSCLC: non‐small cell lung cancer
OS: overall survival
OT: oncologic treatment
PD‐1: programmed cell death protein 1
PD‐L1: programmed death‐ligand 1
PFS: progression‐free survival
PR: partial response
RCC: renal cell carcinoma
SCLC: small cell lung cancer
SD: stable disease
TILs: tumor‐infiltrating lymphocytes
TNBC: triple‐negative breast cancer
UC: urothelial carcinoma

## 1 Introduction

Despite a significant increase in survival rates, breast cancer remains the leading cause of cancer‐related mortality among women worldwide [1]. Among the various biological subtypes, triple‐negative breast cancer (TNBC) is characterized by the lack of expression of estrogen (ER) and progesterone (PgR) receptors, as well as the absence of HER2 overexpression or HER2neu amplification [2]. This specific subtype is known for its poor differentiation, high invasiveness, and increased propensity for both local and distant metastases, leading to a poor prognosis and high recurrence rates. TNBC accounts for ~10%–15% of all breast cancers and is associated with the worst outcomes, with over 50% of patients experiencing relapse within the first 3 to 5 years following diagnosis. In advanced cases, the median overall survival is only 8–13 months [3]. Chemotherapy remains the standard treatment for most metastatic TNBC (mTNBC), although the outcomes are generally poor.

The bad prognosis of TNBC has driven considerable research efforts aimed at identifying novel molecular targets and developing new therapeutic strategies for affected patients [4]. Since breast cancer has traditionally been considered an immunologically “cold” tumor, TNBC is viewed as the most immunogenic subtype of breast cancer due to its molecular features, including higher genetic instability, frequent copy number alterations, and elevated levels of tumor‐infiltrating lymphocytes (TILs) [5, 6]. The potential role of immunotherapy in TNBC, with a particular focus on immune checkpoint inhibitors (ICIs) such as anti‐PD1, anti‐PDL1, and CTLA‐4 antibodies [7, 8] has led to trials exploring ICIs monotherapy, combination of ICIs plus chemotherapy or PARP inhibitors [9], and combination of ICIs agents. Recent reviews have reinforced the role of ICIs as a cornerstone in TNBC management [10].

Currently, the combination of ICIs with chemotherapy is the standard of care in the neoadjuvant setting for patients with stage II or higher TNBC, based on the results of the KEYNOTE‐522 clinical trial. Additionally, in the first‐line setting for PD‐L1‐positive metastatic TNBC, this combination is supported by the outcomes of the KEYNOTE‐355 and IMpassion130 clinical trials [11, 12].

Beyond ICIs, other immunotherapeutic approaches are under investigation, including cancer vaccines, oncolytic viruses, and adoptive cell therapies such as TIL transfer and CAR T‐cell therapy [9, 11, 13]. In this case report, we present two patients with metastatic TNBC who have achieved long‐lasting responses to immunotherapy, and we address the issue of the optimal ICI duration in the case of prolonged response in solid tumors.

## 2 Case Presentation

### 2.1 First Case

In early 2016, during a routine screening examination, a right breast nodule was incidentally discovered in a 60‐year‐old woman in the absence of any clinical symptoms. Subsequently, in July 2016, she underwent a right mastectomy for stage IIa TNBC. Her adjuvant treatment included four cycles of epirubicin plus cyclophosphamide (EC), followed by two cycles of paclitaxel, which were prematurely discontinued due to hypersensitivity reactions.

**FIGURE 1:**
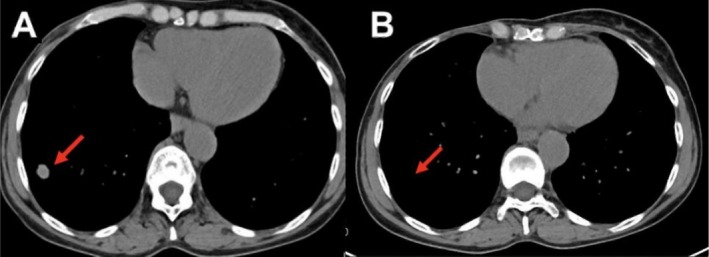
(A) Axial reconstruction of computed tomography scan showing a 15 mm nodule in the right lower lung lobe (March 2019). (B) The same level axial reconstruction in 2020 showed a complete disease response.

Four months after completing adjuvant chemotherapy, the patient developed a histologically confirmed local skin recurrence, considered unsuitable for surgical intervention. She was treated with capecitabine from November 2017 to September 2018, achieving a favorable response that enabled subsequent surgical resection of the skin recurrence. However, disease relapse occurred within 6 months, and by March 2019, metastatic lesions were detected in the liver and lungs (Figure 1A).

The patient was subsequently enrolled in a Phase I clinical trial and initiated therapy with a combination of an anti‐PD‐1 monoclonal antibody (mAb), anti‐LAG‐3 mAb, and anti‐CSF‐1 mAb, administered every 5 weeks, starting April 18, 2019.

The treatment was overall well‐tolerated. The patient experienced a persistent decrease in neutrophil count and mild chronic periorbital edema, primarily attributed to the anti‐CSF‐1 mAb, which was discontinued after six cycles, resulting in full recovery from these adverse effects.

Baseline computed tomography (CT) imaging was performed during screening, followed by radiological assessments every 12 weeks. According to RECIST 1.1 criteria, a complete radiological response was achieved 10 months after the first dose (Figure 1B). The patient remains in complete remission, with no evidence of disease recurrence. She reports a good quality of life without significant late toxicities, and she continues immunotherapy treatment at the time of last follow‐up.

### 2.2 Second Case

**FIGURE 2:**
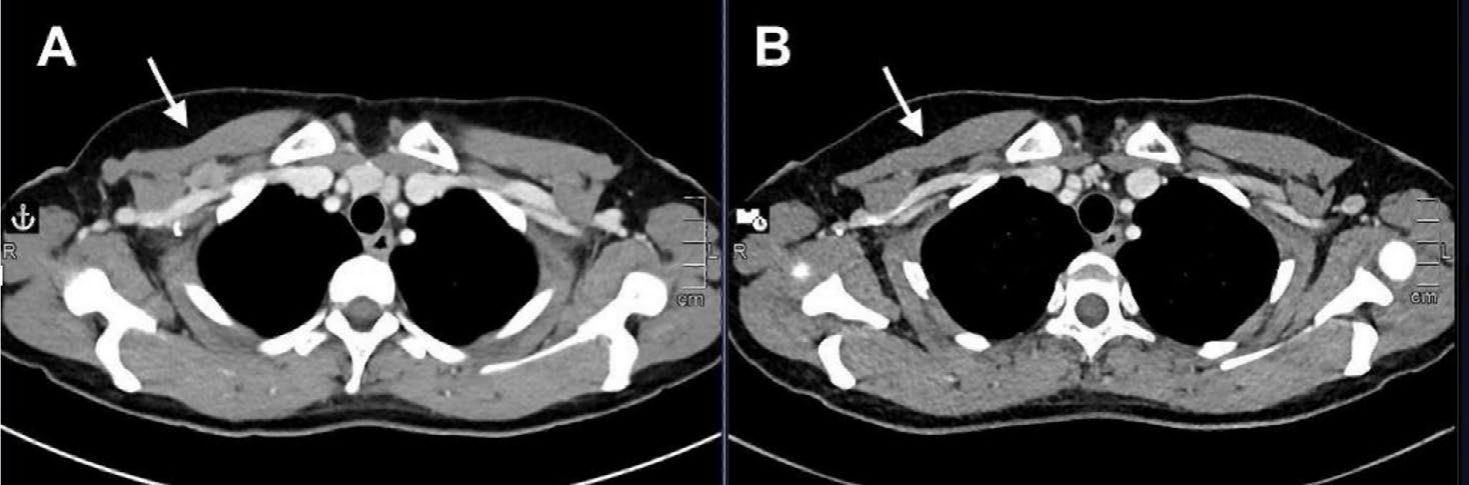
(A) Axial reconstruction of computed tomography scan showing a lymph node conglobate with a size of 20 × 12 mm (June 2019). (B) Corresponding level axial reconstruction showing the complete response.

The second case concerns a 45‐year‐old woman with a congenital BRCA1 mutation. In August 2016, she detected a right breast nodule through self‐examination during auto‐palpation, in the absence of other clinical symptoms. She was subsequently diagnosed with Stage IIa TNBC and underwent bilateral mastectomy, followed by adjuvant chemotherapy consisting of four cycles of EC and twelve cycles of weekly paclitaxel. She completed her adjuvant therapy in March 2017. After a disease‐free interval of 26 months, she developed a histologically confirmed recurrence involving the subclavicular lymph nodes (Figure 2A).

The patient was referred and enrolled in a Phase I clinical trial. She was randomized to receive a combination therapy consisting of avelumab, an anti‐PD‐L1 checkpoint inhibitor (800 mg every 2 weeks), together with the PARP inhibitor talazoparib (1 mg daily). She started treatment on July 22, 2019, and did not experience any significant adverse effects during therapy.

CT scan imaging was conducted prior to treatment initiation, followed by regular radiological assessments. According to RECIST 1.1 criteria, a complete radiological response was achieved 5 months after the first dose (Figure 2B) and has been consistently maintained throughout subsequent assessments. At the last follow‐up visit in March 2025, the patient remains in complete remission and reports a good quality of life without any notable late toxicities. She continues to receive both avelumab and talazoparib, with ongoing treatment well‐tolerated at the time of the last follow‐up.

## 3 Literature Review

To further investigate the presence of reported immunotherapy long responder patients in the metastatic breast cancer setting, we performed a literature review in the most popular medical literature databases (PubMed, Embase, and Google Scholar), with the following criteria: (1) patients affected by metastatic TNBC; (2) patients treated with ICIs; (3) achievement of at least a partial response maintained for a minimum of 5 months. We retrieved a total of 7 reported cases (in addition to the cases presented in our report) [14, 15, 16, 17, 18, 19, 20].

In Table 1 we described the interventions delivered for early‐stage disease; of note only the case reported by Gorshein E. et al. was metastatic since diagnosis. Median age was 59 years. Neoadjuvant therapy was administered in one case, using a combination of 5‐fluorouracil, cyclophosphamide, and epirubicin. Surgical interventions, predominantly mastectomy, were performed in seven cases. Adjuvant therapy was performed in five cases, all comprehensive of a combination of anthracyclines, cyclophosphamide, and taxanes; adjuvant radiotherapy was delivered in two patients (Table 1).

**TABLE 1:** Interventions for the localized stage.

Case	Age	Histology	Stage at diagnosis (NCCN)	Neoadjuvant therapy	Surgery	Adjuvant radiotherapy	Adjuvant therapy
Rivoltini et al.	60	TNBC	IIA	No	Yes	Yes	Yes, doxorubicin + cyclophosphamide + methotrexate + fluorouracil.
Al‐Awadhi A et al.	37	TNBC	IIIB	No	Yes	No	No, due to locoregional recurrence
Al Sayed et al.	49	TNBC	IIIC	Yes, FEC (5‐fluorouracil, Cytoxan, and epirubicin) × 3 plus 3 cycles of docetaxel	Yes	Yes	no
Case one	60	TNBC	IA	No	Yes	Yes	Yes, EC + paclitaxel
Case two	45	TNBC (BRCA1 mut)	IB	No	Yes	No	Yes, EC + paclitaxel
Gorshein E et al.	72	TNBC	IV	No	No		
Fu Y et al.	58	TNBC	IIB	No	Yes	No	Yes, EC + paclitaxel‐carboplatin
Chen et al.	NA	TNBC	II	No	Yes	No	Yes, EC + paclitaxel
Feng et al.	67	TNBC	IIIB	No	Yes	No	Yes, EC + paclitaxel

ICIs were administered as first‐line therapy in 33.3% (3 out of 9) of patients, second‐line therapy in 33.3% (3 out of 9) of patients, and as third or subsequent line of therapy in the other 2 cases (22.2%), most of cases targeting PD1 or PDL1. In two cases anti‐LAG3 were employed. A complete response was achieved in five patients and a partial response in four.

Median progression‐free survival (PFS) was 36 months (range: 8 months to 84 months), and the median disease‐free interval (DFI) was 60 months (range: 15 months to 84 months). Notably, 55.5% (5 out of 9) of patients continued ICI therapy at the time of data analysis. In 22.2% of patients (2 out of 9), immunotherapy treatment was stopped after 2 years, both cases without progression after a follow‐up of 12 and 20 months, respectively. All patients, except two (who progressed after 20 and 8 months, respectively, of sustained partial response), were alive at the time of case publication.

## 4 Discussion

The two cases presented herein refer to patients affected by Stage IV TNBC with a story of long response to ICI treatment, a well‐known uncommon, and favorable situation alongside the therapeutic pathway of this type of tumor.

Both patients received immunotherapy as an early line for metastatic disease; although the disease burden was limited, PFSs are remarkably higher when compared to previously published data summarized in Table 2: among the four cases reaching complete remission, only the one provided by Rivoltini et al. presents a better PFS [14] (Table 2).

**TABLE 2:** ICIs utilization and outcomes of the retrieved cases.

Case	Systemic‐metastatic therapies before ICI	PDL1	Involvement sites at the beginning of OT	Type of ICIs	Line of treatment with ICI	Best‐response	PFS	DFI	Continuing ICIs (yes vs. no)	Alive (yes vs. no)
Rivoltini et al.	NA	No	Chest wall skin	Anti PD1 + Anti LAG3	Ninth	CR	84 months	84 months	Yes	Yes
Al‐Awadhi A et al.	No	1.5% (IHC—Ventana Ab SP142)	Lung + right chest wall	Atezolizumab (anti‐PDL1) + Nab‐paclitaxel	First	CR	36 months	36 months	No, stopped after 2 years	Yes
Al Sayed et al.	Yes (capecitabine)	20% (IHC)	Lymphnodes, lung, bones, soft tissue	Durvalumab (anti‐PDL1) + paclitaxel	Second	CR	15 months	15 months	Yes	Yes
Case one	Yes (capecitabine)	Negative (CPS)	Lung	Anti PD1 + anti LAG 3 + anti CSF1	Second	CR	60 months	60 months	Yes	Yes
Case two	No	10% (CPS)	Lymphnodes	Anti PD1 + PARP inhibitor	First	CR	60 months	60 months	Yes	Yes
Gorshein E et al.	No	Positive (other information NA)	Bones, lung, lymphnodes	Pembrolizumab (anti PD1)	First	PR	44 months	/	No, stopped after 2 years	Yes
Fu Y et al.	Yes (anlotinib + gemcitabine)	< 1% CPS score	Lung		Third (*Relapse within 6 months after adjuvant therapy)	PR	8 months	/	Yes	Yes
Chen et al.	Yes, not further information available	NA	Lung, pleura	Anti PD1 (sintilimab), and anlotinib plus Pirarubicin	Second	PR	20 months	/	/	No
Feng et al.		NA	Liver	Atezolizumab (anti‐PDL1) + Nab‐paclitaxel	Third and fourth (only fourth with a PFS > 5 month)	PR	9 months	/	/	No

Several factors support TNBC as a good candidate for immunotherapy, including the high presence of tumor‐infiltrating immune cells, the generation of neoantigens due to the tumor's mutational load, and the high expression of PD‐1 and PD‐L1 (70% and 59% of cases, respectively, in TNBC).

**FIGURE 3:**
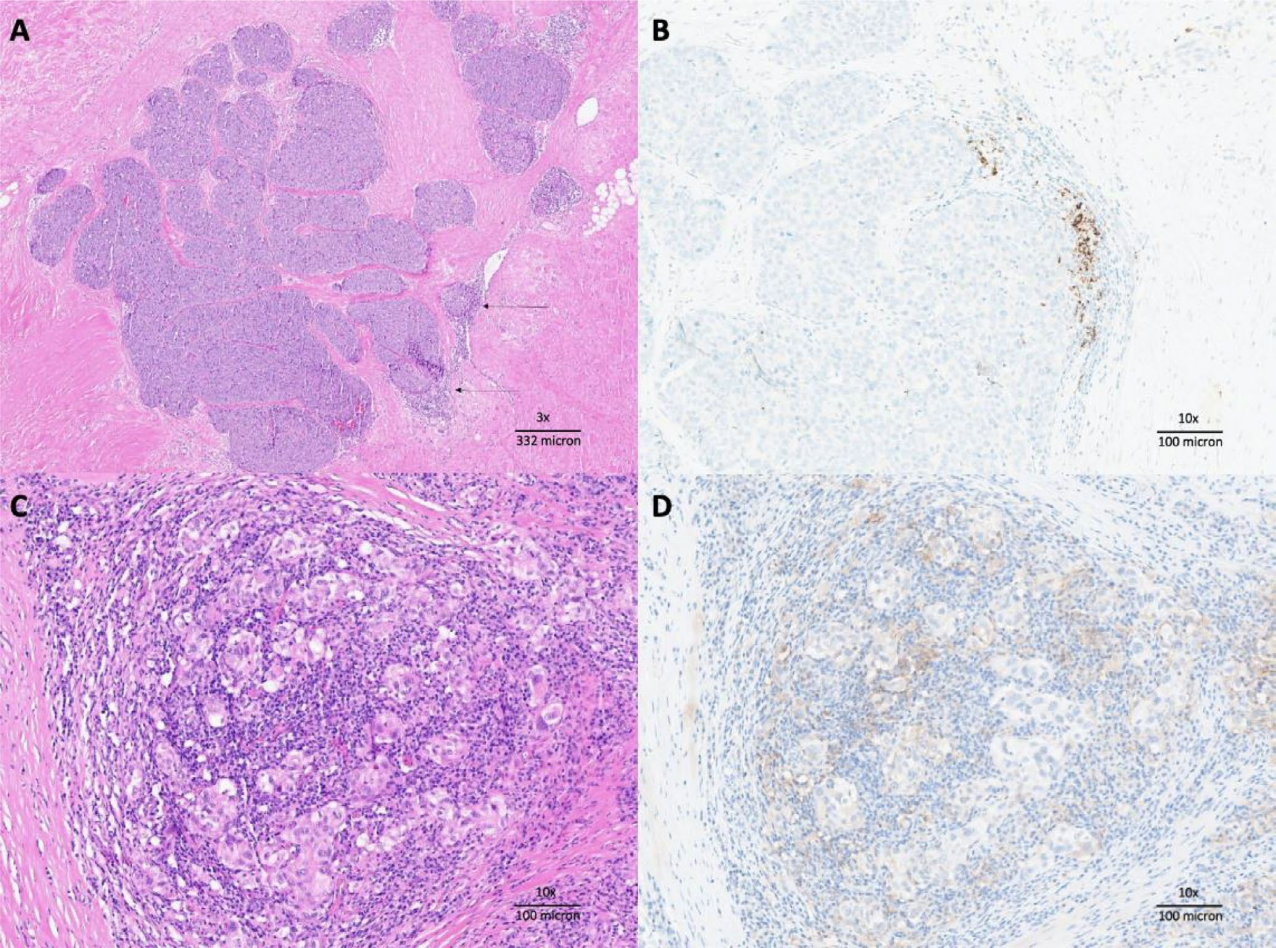
(A) First case described: The carcinoma metastasis grows in solid nests in a fibrous stroma, with minimal associated immune infiltrate (arrow) (×10). (B) First case described: PD‐L1 IHC, clone SP142, ×10: The immune score (IC) is > 1%, the PD‐L1‐positive cells are immune cells. CPS score turned out to be negative. (C) Second case described: Figure shows carcinoma metastasis in the form of isolated elements within the context of the lymph node parenchyma (×10). (D) Second case described: PD‐L1 IHC, clone 22C3, ×10: The combined positive score (CPS) is 10%; note that the PD‐L1‐positive neoplastic cells are larger than the stained immune cells.

Notably, ~80% of TNBC tumors exhibit significant infiltration by tumor‐infiltrating lymphocytes (TILs). However, the contrasting TIL levels in the cases described (5% and 80%, respectively) as well as PD‐L1 expression levels (negative in the first case and 10%—CPS score—in the second one) exemplify the heterogeneity of the immune microenvironment in TNBC and its variable impact on clinical outcomes [21, 22, 23] (Figure 3A–D). Recent data from the ENHANCE‐1 trial also underlined how spatial biomarkers of the tumor microenvironment may influence ICI response in TNBCB [24]. The elapsed time to achieve a complete response was variable in our cases: 10 versus 5 months.

Evidence from previous studies has established higher PD‐L1 expressions and TIL levels, particularly stromal TILs (sTILs), as robust prognostic markers associated with improved DFS and OS, as well as enhanced response to chemotherapy and immunotherapy. However, the exceptional long‐term response observed in the case with only 5% TILs and negative PD‐L1 suggests that other, less explored mechanisms, such as neoantigen quality or non‐TIL‐dependent immune pathways, may play pivotal roles in driving sustained therapeutic efficacy [22]. This reinforces the need to refine the integration of PD‐L1 and TILs as biomarkers, potentially incorporating them into multiparametric models that account for the interplay between intrinsic tumor biology and host immunity, to better predict outcomes and guide treatment decisions in TNBC [22, 25].

In the second case presented, a key factor may have contributed to the tumor response. Indeed, it is well established that combining immunotherapy with standard oncological treatments can modify the tumor microenvironment, leading to an improved therapeutic response. Preclinical research has demonstrated that the combination of PARP inhibitors with PD‐L1 pathway blockade can significantly enhance therapeutic outcomes, as evidenced by the presented data [9].

Moreover, early‐phase clinical studies support this hypothesis: the TOPACIO/KEYNOTE‐162 trial of niraparib plus pembrolizumab reported an ORR of ~21% with durable responses in some BRCA1/2‐mutated patients, the MEDIOLA study of olaparib plus durvalumab showed high disease control in BRCA‐mutated breast cancer, and the JAVELIN BRCA/ATM trial of avelumab plus talazoparib demonstrated antitumor activity in BRCA1/2‐ or ATM‐altered tumors [26, 27, 28].

Given this, the question of the optimal duration of ICI treatment in cases of durable response should be raised, especially considering the limited number of long‐term responders to ICIs in TNBC cases reported in the literature. Notably, in the TNBC cases described by Al‐Awadhi and Gorshein, immunotherapy was discontinued after 2 years, with the responses (partial and complete, respectively) sustained until the publication of the articles [15, 17].

Moving to other tumors, a recent review examined early discontinuation of immunotherapy in metastatic melanoma patients. The review reported that in the KEYNOTE001 trial, 90% of IT responses persisted post‐treatment discontinuation. Similarly, in the KEYNOTE006 trial, 76% of patients who achieved complete response maintained it after stopping treatment, and notably 8% of those with partial response converted to complete response after IT discontinuation [29].

Furthermore, a recent meta‐analysis aimed to determine the optimal duration of immune checkpoint inhibitors (ICIs) in solid tumors. The analysis reviewed 57 studies involving 22 977 patients with various solid tumors, including melanoma, non‐small cell lung cancer (NSCLC), small cell lung cancer (SCLC), head and neck squamous cell carcinoma (HNSCC), renal cell carcinoma (RCC), and urothelial carcinoma (UC). The findings indicated that in melanoma patients, prolonged ICI administration enhances overall survival compared to treatment limited to 2 years. Conversely, in NSCLC patients, combining ICI with standard therapy (SoC) for 2 years yielded better survival outcomes than extended treatment. No further significant differences were observed among other tumor types. The review concluded that the optimal ICI treatment duration varies by tumor type; thus, it makes it difficult to conclude a clinical behavior common to different tumor types [30].

Randomized trials are currently ongoing to address the issue (Table 3). Unfortunately, most of these studies focus on melanoma, with only one considering other tumors, also including TNBC patients (NCT04157985).

**TABLE 3:** Ongoing randomized clinical trials aiming to describe optimal ICIs duration.

Identification number	Treatment to discontinue	Design	Involved histologies	Recruiting (yes vs. no)	Primary endpoint
NCT04157985	PD‐1/PD‐L1 inhibitor	Randomized. 1:1 randomization at 1 year between stop or continue treatment, if a response has been achieved.	NSCLC, bladder, HNSCC, renal, melanoma, cervical, Merkel cell, MMR/MSI (colon, rectal, cholangio‐carcinoma, esophageal, ovarian, uterine), anal, gastric and GE junction, hepatocellular, triple negative breast cancer	Yes	PFS
NCT02821013	PD‐1 inhibitor	Randomized. STOP treatment when MTR. Resume treatment at progression.	Melanoma	Yes	OS
ISRCTN15837212	PD‐1 inhibitor	Randomized. 1:1 randomization at 1 year if CR, PR, SD between stop or continue treatment.	Melanoma	Yes	PFS
NCT04462406	PD‐1 inhibitor	Randomized. STOP treatment at one year if PET negative or positive with negative biopsy.	Melanoma	Yes	Event‐free survival
NCT05652673	PD‐1 inhibitor + CTLA‐4 inhibitor	Single arm. STOP treatment if CR or ongoing PR.	Melanoma	Yes	Response rate

Future studies will also explore biological, molecular predictive factors, as well as radiomic ones to identify patients with a lower likelihood of disease recurrence after treatment discontinuation (NCT04462406).

Of note, a recent survey on metastatic urothelial cancer showed that the effectiveness of immunotherapy could be predicted as early as 3 weeks into treatment by analyzing cancer DNA in the blood using liquid biopsy. Such approaches could help facilitate timely adjustments to immunotherapy duration in urothelial cancer patients [31].

In conclusion, due to the lack of strong evidence, clinical decisions on ICIs treatment should carefully consider patient treatment tolerance and the presence and severity of side effects when assessing the risk–benefit balance. This is especially important when considering whether to continue treatment beyond 2 years in cases where at least disease stability has been achieved. Prospective trials addressing this endpoint should be designed to warrant more appropriate choices in decision‐making.

## 5 Conclusions

This paper reports two exceptional cases of long‐term complete response to immune checkpoint inhibitors in metastatic TNBC, including one patient with low TILs and PD‐L1 negativity—features typically associated with poor immunotherapy outcomes. Both patients achieved durable responses exceeding 5 years following ICI‐based treatment, an extremely rare occurrence in this setting, with only a few similar cases reported in the literature. Notably, none of the previously described cases exhibited such prolonged benefit, highlighting the scientific relevance of reporting these exceptional outcomes.

The findings underscore the heterogeneity of TNBC's immune landscape and suggest that current biomarkers, such as TILs and PD‐L1 expression, are insufficient to fully predict the benefit from immunotherapy. This represents an important consideration emerging from the study, emphasizing the need for improved predictive models. Moreover, this report underlines the current gap in evidence regarding the optimal duration of ICIs in long responders. A further key message is the need to individualize treatment duration, carefully balancing clinical response, tolerability, and the use of emerging predictive tools, such as circulating tumor DNA, radiomic analyses, and gene expression signatures, particularly in the absence of strong prospective data.

## Author Contributions

Conceptualization: Simone Rota, Silvia Damian. Data curation: Simone Rota, Laura Cattaneo, Chiara Volpi, Alessandra Casale, Sara Cresta. Formal analysis: Simone Rota, Chiara Volpi, Sara Cresta. Funding acquisition: Simone Rota. Investigation: Simone Rota, Matteo Duca. Methodology: Simone Rota. Project administration: Simone Rota. Resources: Simone Rota, Laura Cattaneo, Alessandra Casale, Sara Cresta. Software: Sara Cresta. Supervision: Filippo De Braud, Sara Cresta. Validation: Simone Rota, Giorgia Villa, Elisabella Ebrahem, Diletta Sorrentino, Antonia Martinetti. Visualization: Simone Rota, Matteo De Monte, Laura Cattaneo, Alessandra Casale, Sara Pessina, Sara Cresta. Writing – original draft: Simone Rota, Carolina Sciortino, Sara Cresta. Writing – review and editing: Simone Rota, Carolina Sciortino.

## Ethics Statement

The authors have nothing to report.

## Consent

Informed consent was obtained from both patients for the publication of clinical data and images. Signed consent forms have been archived and uploaded among the supplementary files attached to this manuscript.

## Conflicts of Interest

Filippo De Braud reported receiving personal fees from Bristol Myers Squibb, Roche, Merck, Bayer, Ignyta, Dephaforum, Biotechespert, Prime Oncology, Pfizer, Nadirex, Ambrosetti, Incyte, Motore Sanità, Fare Comunicazione, Itanet, European School of Oncology, Accmed, Idea‐z, Dynamicom Education, Pierre Fabre, Mattioli 1885, MCCann Health, MSD, IQVIA, Celgene, Amgen, and Sanofi; grants from Novartis, Roche, Bristol Myers Squibb, Celgene, Incyte, Nerviano Medical Sciences, Merck, Darmstadt, Kymab, Pfizer, Tesaro, and Kenilworth; serving on advisory boards for Tiziana Life Sciences, Bristol Myers Squibb, Celgene, Novartis, Servier, Pharm Research Associated, Daiichi Sankyo, Ignyta, Amgen, Pfizer, Octimet Oncology, Incyte, Pierre Fabre, Eli Lilly, Roche, AstraZeneca, Gentili, Dephaforum, Merck, Kenilworth, Bayer, Fondazione Menarini, Sanofi, Taiho; serving as principal investigator for studies by Novartis Farma, AstraZeneca, F. Hoffmann‐La Roche, Bristol Myers Squibb, AnHeart Therapeutics, and Apollomics outside the submitted work. Other authors declare no conflicts of interest.

## Data Availability

Authors confirm that data supporting this case report, and the associated literature review are available within the article.
